# Integrating Blood Collection within Household Surveys: Lessons Learned from Nesting a Measles and Rubella Serological Survey within a Post-Campaign Coverage Evaluation Survey in Southern Province, Zambia

**DOI:** 10.4269/ajtmh.18-0320

**Published:** 2018-10-01

**Authors:** Simon Mutembo, Andrea Carcelen, Francis D. Mwansa, Kelly Searle, Jane W. Wanyiri, Chris Book, Philip E. Thuma, William J. Moss, Kyla Hayford

**Affiliations:** 1 Ministry of Health, Government of the Republic of Zambia, Lusaka, Zambia;; 2 Department of Epidemiology and Biostatistics, University of Georgia, College of Public Health, Athens, Georgia;; 3 International Vaccine Access Center, Department of International Health, Johns Hopkins Bloomberg School of Public Health, Baltimore, Maryland;; 4 Department of Epidemiology, Johns Hopkins Bloomberg School of Public Health, Baltimore, Maryland;; 5 Macha Research Trust, Choma, Zambia

## Abstract

Age-specific population immunity to many vaccine-preventable diseases can be measured using serological surveys. However, stand-alone serological surveys are infrequently conducted in low- and middle-income countries because of costs, operational challenges, and potential high refusal rates for blood collection. Nesting a serosurvey within a household cluster survey may overcome some of these challenges. We share lessons learned from nesting a serosurvey within a measles and rubella vaccination post-campaign coverage evaluation survey (PCES). In 15 of the 26 PCES clusters in Southern Province, Zambia, we collected dried blood spots from 581 participants aged 9 months and older. Household participation rates for the main PCES were higher in the serosurvey clusters (86%) than PCES-only clusters (71%), suggesting that a serosurvey can be successfully integrated without adversely affecting PCES participation. Among households that participated in the PCES, 80% also participated in the serosurvey and 86% of individuals available in the household provided a blood sample for the serosurvey. Substantial planning and coordination, additional staff training, and community mobilization were critical to the success of the serosurvey. Most challenges stemmed from using different data collecting tools and teams for the serosurvey and PCES. A more efficient design would be to fully integrate the serosurvey by adding blood collection and additional questions to the PCES.

Age-specific population immunity, measured as the proportion of individuals across age strata with protective levels of antigen-specific immunoglobulin G antibodies, can be estimated using serological surveys for many vaccine-preventable diseases (VPDs).^1^ However, serosurveys are infrequently conducted in low- and middle-income countries because of cost, operational challenges to blood collection, transport and processing of samples, and concerns about participant refusal rates for blood collection.^2–5^ Instead, specific population immunity is commonly inferred from vaccination coverage estimates, which may be inaccurate and do not reflect true population immunity because of primary and secondary vaccine failure and exposure to natural infection.^2,5,6^ Rigorously designed serosurveys directly measure population susceptibility and immunity and can be used to assess the proportion of children protected after a vaccination campaign.^1^ Knowledge of age-specific immunity profiles, spatial clustering, and susceptibility among subpopulations can guide targeted vaccination activities and improve the efficiency of immunization programs.^7,8^

For serological surveillance of VPDs to be practical and sustainable, serosurveys should ideally be conducted within existing surveillance systems or multipurpose household surveys, such as Demographic and Health Surveys or Multiple Indicator Cluster Surveys, nationally representative surveys for malaria or human immunodeficiency virus infection, or post-campaign coverage evaluation surveys (PCES).^9^ We nested a serological survey within a PCES in one province of southern Zambia following a national measles and rubella (MR) vaccination campaign. We describe the processes that enabled successful implementation of the nested serosurvey and highlight the challenges and lessons learned.

The national catch-up MR vaccination campaign was conducted in Zambia from September 19 to 24, 2016. The campaign targeted children aged 9 months to 15 years, and it was the first time a rubella-containing vaccine was administered nationally in Zambia. Two months after the MR campaign, the Ministry of Health (MoH) commissioned a PCES, a nationwide household cluster survey aimed at measuring vaccination coverage achieved by the campaign and routine vaccination.^10^ Data collection for the PCES was conducted between November 21 and December 3, 2016. The PCES used a two-stage cluster survey design adapted from the WHO Vaccination Coverage Cluster Survey Manual to select 30 clusters per province with probability of cluster selection proportional to estimated size and 12 households per cluster.^11^

To measure age-specific population immunity to MR viruses, the serosurvey team partnered with the PCES team to conduct a serological survey using dried blood spots (DBS) in Southern Province, Zambia. Although the PCES was conducted throughout the country, the nested serosurvey was conducted only in the Southern Province to leverage access to a laboratory with storage facilities and expertise in serology. Measles and rubella were the primary antigens of interest for both the PCES and serosurvey, but the target age ranges differed. The PCES focused on children eligible for the vaccination campaign but the serosurvey aimed to evaluate population immunity among children aged 9 months and older as well as adults, including rubella immunity among women of childbearing age. The sampling strategy for the serosurvey was based on the design of the PCES and aimed to estimate seroprevalence with a precision of ±5%. We assumed a 36% nonparticipation rate for blood collection based on a previous serosurvey.^12,13^

For logistical reasons, the serosurvey was conducted in a random sample of 15 PCES clusters in the Southern Province. Overall, 249 of the 312 eligible households were enrolled in the PCES survey in the Southern Province (Figure 1). The serosurvey did not appear to impact household participation in the PCES as 71% of households in the PCES-only clusters participated, compared with 86% of households in the PCES plus serosurvey clusters. For the serosurvey, 124 (80%) PCES households were enrolled over 12 days. Of those households that did not participate in the serosurvey, 8% were not available and 12% refused. Reasons for refusal were different among households but were mostly because of nonavailability of the head of the household at the time of the serosurvey. One cluster, which represented 12 households, refused to take part in both the PCES and the serosurvey because the community was suspicious that personal information and blood would be used for satanic religious purposes. Combining the PCES and serosurvey participation rates, 69% of households eligible in the PCES sampling frame were included in the serosurvey.

**Figure 1.**
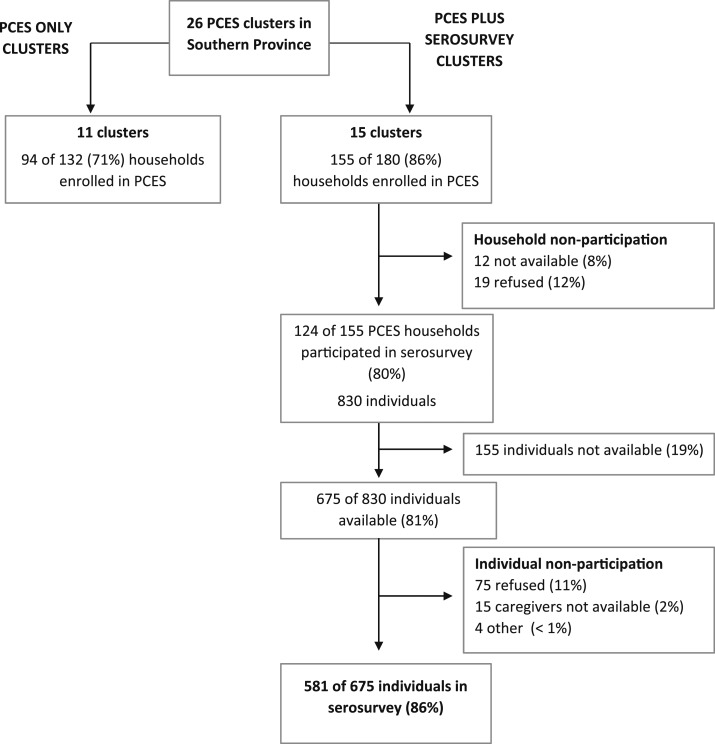
CONSORT enrollment flow diagram comparing post-campaign coverage evaluation survey (PCES) plus serosurvey clusters and PCES-only clusters in Southern Province, Zambia.

Within the 124 households that agreed to participate in the serosurvey, 830 individuals were eligible and 675 (81%) of them were available at the time of the serosurvey. Of the available individuals, 581 (86%) participated and provided a blood sample, 11% refused, 2% did not have caregiver available to provide consent, and 1% were ineligible for other reasons.

Planning and coordination were required at all levels and throughout each stage of the survey. A technical coordination group comprising MoH officials, WHO Zambia officials, and PCES implementing partners worked with the serosurvey planning team for 3 months before the PCES to develop a coordination plan for the two surveys. The serosurvey protocol was developed through a consultative process and sought to minimize disruption to the PCES. For example, two separate electronic questionnaires for the PCES and serosurvey were used. To avoid repeating questions, data from the PCES questionnaire were shared with the serosurvey team. Additional questions not captured by the PCES were asked in the serosurvey questionnaire after the PCES team completed their questionnaire.

In anticipation of hesitancy toward blood collection, the serosurvey team developed a sensitization plan at the provincial, district, and community levels in collaboration with MoH district management teams aimed at communicating the purpose and procedures of the serosurvey to key stakeholders in study communities. The plan was adapted from previous household surveys that collected blood in the community and achieved good participation rates.^12,14^ A letter from the MoH was issued to the provincial and district medical officers to inform staff about the serosurvey in their communities. Within each district, the district health management teams implemented community sensitization based on micro-plans developed for each study community. Typically, the nurse in charge, the environmental health technician at health facilities in the study communities, and a community representative, such as a community health worker, conducted door-to-door sensitization at least 3 days before the survey and made announcements on the local radio station. They facilitated meetings between the field team and the local community leaders to introduce the serosurvey, and community representatives accompanied the serosurvey teams to all households during data collection, ensuring continuity between the sensitization and serosurvey field activities.

Each serosurvey field team comprised four data collectors paired with two PCES data collectors. At least one of the four serosurvey data collectors was a certified clinical staff trained in collecting DBS and one of the two PCES data collectors was the team leader. The serosurvey teams were trained for 5 days in eliciting immunization history, reviewing household-based immunization records, informed consent process, fingerprick blood collection, and DBS preparation. Collection of fingerprick blood on DBS filter paper provided an advantage because it does not require staff with phlebotomy skills, cold chain, or access to a centrifuge in the field, which are common obstacles to blood collection in the field.^15^

Two separate ethical approvals were obtained for the serosurvey and the PCES because serosurvey participants were required to provide written informed consent. Ethical approval for the serosurvey was obtained from Johns Hopkins Bloomberg School of Public Health Institutional Review Board, Macha Research Trust Ethics Review Committee, and the Zambia National Health Research Regulatory Authority. Enrollment of all household members, including those outside the target age group for the PCES, increased the length of interviews and moderately slowed data collection. On average, data collection took 2 days in a PCES-only cluster and 3 days in a paired PCES and serosurvey cluster.

Most challenges stemmed from the fact that two separate electronic questionnaires were administered: one for the PCES questions and the other for additional questions and procedures relevant to the serosurvey (Figure 2). In addition, the serosurvey was a research project and, therefore, required additional ethical approvals, informed consent processes, separate data collection tools, and linkage of household and participant identification numbers during data analysis to PCES data. A more efficient design would be to fully integrate the serosurvey by adding blood collection and additional questions to the PCES. This approach was not implemented because of limited time in the planning phase, uncertainties regarding the timeline and survey instruments, and the concern expressed by the PCES leadership that adding blood collection could increase refusals and induce a response bias for the main survey. The participation rates for the PCES were higher in the serosurvey clusters than those for PCES-only clusters, and individual refusals for blood collection were low although some hesitancy was observed. Future surveys need to weigh the risks of refusal and its potential impact on representativeness against the efficiency, cost savings, and benefits of fully integrating blood collection into PCES activities.

**Figure 2.**
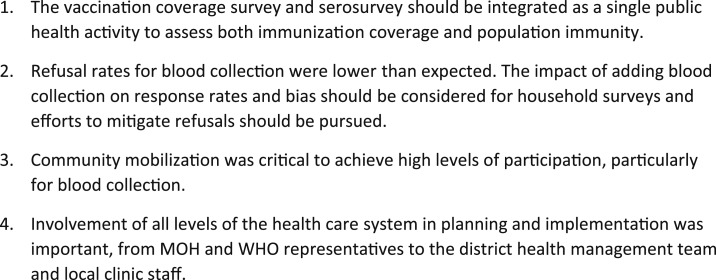
Lessons learned from nesting a serosurvey within a vaccination coverage survey. MoH = Ministry of Health.

By successfully conducting a serological survey within the PCES in Southern Province, Zambia, we demonstrated the feasibility of nesting a serosurvey in a health-related household survey. With appropriate statistical and logistical adaptations, serological surveys can be implemented as part of other planned household surveys, providing a platform for blood collection to measure antibodies to vaccine-preventable and emerging infectious disease antigens while leveraging existing surveillance systems and resources. Lessons learned from this study provide guidance for implementation of future nested serosurveys (Figure 2).
